# Liposome-Based Adjuvants for Subunit Vaccines: Formulation Strategies for Subunit Antigens and Immunostimulators

**DOI:** 10.3390/pharmaceutics8010007

**Published:** 2016-03-10

**Authors:** Signe Tandrup Schmidt, Camilla Foged, Karen Smith Korsholm, Thomas Rades, Dennis Christensen

**Affiliations:** 1 Department of Pharmacy, Faculty of Health and Medical Sciences, University of Copenhagen, Universitetsparken 2, 2100 Copenhagen Ø, Denmark; sxs@ssi.dk (S.T.S.); Camilla.foged@sund.ku.dk (C.F.); Thomas.rades@sund.ku.dk (T.R.); 2 Department of Infectious Disease Immunology, Statens Serum Institut, Artillerivej 5, 2300 Copenhagen S, Denmark; kkn@ssi.dk

**Keywords:** liposome, vaccine adjuvant, formulation, immunostimulator, antigen

## Abstract

The development of subunit vaccines has become very attractive in recent years due to their superior safety profiles as compared to traditional vaccines based on live attenuated or whole inactivated pathogens, and there is an unmet medical need for improved vaccines and vaccines against pathogens for which no effective vaccines exist. The subunit vaccine technology exploits pathogen subunits as antigens, e.g., recombinant proteins or synthetic peptides, allowing for highly specific immune responses against the pathogens. However, such antigens are usually not sufficiently immunogenic to induce protective immunity, and they are often combined with adjuvants to ensure robust immune responses. Adjuvants are capable of enhancing and/or modulating immune responses by exposing antigens to antigen-presenting cells (APCs) concomitantly with conferring immune activation signals. Few adjuvant systems have been licensed for use in human vaccines, and they mainly stimulate humoral immunity. Thus, there is an unmet demand for the development of safe and efficient adjuvant systems that can also stimulate cell-mediated immunity (CMI). Adjuvants constitute a heterogeneous group of compounds, which can broadly be classified into delivery systems or immunostimulators. Liposomes are versatile delivery systems for antigens, and they can carefully be customized towards desired immune profiles by combining them with immunostimulators and optimizing their composition, physicochemical properties and antigen-loading mode. Immunostimulators represent highly diverse classes of molecules, e.g., lipids, nucleic acids, proteins and peptides, and they are ligands for pattern-recognition receptors (PRRs), which are differentially expressed on APC subsets. Different formulation strategies might thus be required for incorporation of immunostimulators and antigens, respectively, into liposomes, and the choice of immunostimulator should ideally be based on knowledge regarding the specific PRR expression profile of the target APCs. Here, we review state-of-the-art formulation approaches employed for the inclusion of immunostimulators and subunit antigens into liposome dispersion and their optimization towards robust vaccine formulations.

## 1. Introduction

Together with improved hygiene and antibiotics, vaccines have provided the most important contribution to the reduction of the annual number of deaths caused by infectious diseases, which have affected mankind throughout history [1]. The majority of the currently marketed vaccines are directed against pathogens with little antigen variability and for which infection can be effectively prevented by neutralizing or opsonizing pathogen-specific antibody responses [2]. However, several pathogens fall outside this category, and novel vaccines are therefore required, which can effectively and safely prevent their infection [3].

A modern strategy for designing novel vaccines is the subunit vaccine strategy. The introduction of the reverse vaccinology approach and technological advances in the manufacturing of subunit antigens have altogether enabled rational selection and production of the specific epitopes needed for efficacious pathogen-specific vaccines. The antigens selected for subunit vaccines are highly purified and thus show reduced reactogenicity due to the absence of exogenous immune-activating components as compared to, e.g., the whole-cell vaccines [4]. Therefore, the increased safety also comes at the price of reduced immunogenicity, and adjuvants are usually added to provide the necessary innate immunopotentiation and to direct the desired adaptive immune response [5,6]. Adjuvants are generally defined as functional excipients and constitute a heterogeneous group of compounds, which can broadly be classified into delivery systems or immunostimulators, with many adjuvants possessing both properties. Delivery systems, which serve as the carriers of the antigen and the immunostimulators in the vaccine, are often particles, e.g., liposomes, emulsion droplets and immune-stimulating complexes (ISCOMs) [7,8]. The co-delivery of antigen and immunostimulators, which are ligands for pattern-recognition receptors (PPRs) expressed by antigen-presenting cells (APCs) in the immune system, has been shown to be required for stimulating protective immune responses due to the need for concomitant antigen presentation and activation of APCs [9,10,11,12]. Liposome-based adjuvants may act as both delivery systems for subunit antigens and as immunopotentiators, and they are highly versatile adjuvants, as they can be tailored through (i) the choice of lipid composition, (ii) the inclusion of immunostimulating compounds, (iii) the choice of formulation method and (iv) the mode of antigen and immunostimulator association.

Liposomes, formed by self-assembly upon dispersion of certain amphiphilic lipids in aqueous buffer, were initially utilized as model membranes [13,14]. The use of liposomes as vaccine adjuvants was explored already in 1974, where it was shown that immunization of mice with diphtheria toxoid (DT) adjuvanted with phospholipid-based liposomes resulted in increased antibody titers as compared to immunization with non-adjuvanted DT [15]. The use of liposomes as vaccine adjuvants has been intensively investigated and clinically tested following these studies [16,17].

Amphiphilic lipids with a cylindrical shape tend to form lamellar phases, which upon equilibration with excess water may form closed vesicles that are usually composed of several lipid bilayers separated by aqueous layers (multilamellar liposomes) [14]. The lipid composition of the liposomes and the preparation method determine important physicochemical characteristics of the vesicles, e.g., the particle size, membrane fluidity, hydrophobicity and surface charge. Thus, the physicochemical properties of liposome dispersions can be controlled via their composition and preparation method [18,19,20,21,22,23,24]. The composition of the liposomes might also influence the incorporation strategies chosen for other molecules, such as immunostimulators and subunit antigens, as well as determine the type and strength of immune response induced by the vaccine [17]. The adjuvant mechanism of liposomes is characterized by their ability to interact with APCs, and enhance the exposure of antigen and immunostimulators to the APCs [25]. When using liposomes as adjuvants, they therefore act as delivery systems for the antigen and the immunostimulators. The versatility of liposomes also has the benefit that it is possible to incorporate different types of molecules into the same liposome dispersion, e.g., a lipid-based immunostimulator and a protein-based antigen. In this review, we discuss the effect of key physicochemical properties of liposome-based adjuvants on the immune responses they induce and incorporation strategies employed for immunostimulators and antigens.

## 2. The Physicochemical Properties of Liposomes Affect the Immune Responses

The physicochemical characteristics of liposomes may have a significant impact on the immune responses, and they are often decisive for which antigen and/or immunostimulator incorporation strategies can be used. In the following, the important physicochemical characteristics will be discussed: particle size, surface charge, surface modification and membrane fluidity.

### 2.1. The Effects of Particle Size

Several studies suggest that the average particle size of the liposomes influences the type of immune response that is induced upon immunization [18,26]. The particle size may influence the draining kinetics of liposomes, as smaller-sized liposomes have been shown to be cleared faster from the site of injection (SOI) than larger-sized liposomes [27,28]. For example, the retention at the SOI following subcutaneous (s.c.) immunization was inversely dependent on the particle size for liquid-crystalline state egg-phosphatidylcholine:egg-phosphatidylglycerol:cholesterol (EPC:EPG:Chol)-liposomes (average particle sizes: 40, 70, 170, 400 nm and larger non-sized) and dioleoyl phosphoethanolamine:dioleoyl-trimethylammonium propane (DOPE:DOTAP):EPC-liposomes (average particle sizes: 140 and 560 nm, polydispersity index (PDI): 0.14 and 0.55, respectively). Thus, a significantly bigger fraction of large-sized liposomes was recovered at the SOI, as compared to smaller-sized liposomes [27,28]. However, no size dependency was observed on the recovery of liposomes in the draining lymph nodes over a period of 52 h and 8 days post-injection, respectively [27,28]. This suggests that the larger-sized liposomes, that do escape the SOI, are more effectively retained in the lymph nodes by, e.g., phagocytosis by innate cells, than the smaller-sized liposomes, which pass on to the blood [27]. The results of these studies are ambiguous, as it has to be taken into account that the effect of particle size on the immune response is also dependent on other factors, such as the administration route and the lipid composition. In addition, it should be kept in mind that the particle sizes given for the liposomes are the average of distributions. As a consequence, a sample with a heterogeneous particle size distribution might contain particles in a size range that rapidly escapes the SOI, as well as particles that are trapped at the SOI.

The particle size of liposomes possibly influences their biodistribution, thus affecting the induced immune response. Aseptically-prepared, endotoxin-free 1-monopalmitoyl glycerol:dicetyl phosphate (DCP):Chol liposomes at 560 nm, administered s.c. with model antigen chicken egg ovalbumin (OVA), induced strong interferon (IFN)-γ responses, while liposomes at 155 nm induced an IL-5 response. The interpretation of this was that larger-sized liposomes preferentially induce a T-helper cell (Th)-1 response, which is shifted to a Th2 response for smaller liposomes [26]. Similarly, dipalmitoyl phosphatidylcholine (DPPC):Chol-liposomes with an average particle size of 400, 1000 and 1100 nm (PDI = 0.37, 0.73 and 1.00, respectively) induce a stronger Th1 response than liposomes with an average particle size of 120 nm (PDI = 0.09) following s.c. immunization [18]. However, no dependency of particle size on the immune response was observed in a study with solid-ordered, gel state, cationic dioctadecyldimethylammonium bromide:trehalose-6,6´-dibehenate (DDA:TDB)-liposomes, also called CAF01, when comparing liposomes with approximate sizes of 200, 700, 1500 and 2500 nm. All vaccine formulations were administered intramuscularly (i.m.) and stimulated a Th1 response [19]. This is likely due to the cationic surface charge, which causes even small liposomes to form a depot at the SOI, due to interaction with, e.g., negatively-charged interstitial proteins, as discussed below.

### 2.2. Effect of Surface Charge

The apparent surface charge of liposomes is primarily determined by the composition of the lipids in the liposome bilayers. The surface charge can be modified by using charged lipids in the liposomes, e.g., positively-charged acetylated ammonium compounds diacyl-dimethylammonium-propane (DAP), diacyl-trimethylammonium-propane (TAP) and dimethyldiacylammonium compounds (DDA), stearylamine (SA), negatively-charged phosphatidylserine (PS) or phosphatidic acid (PA) compounds [20,21,22,29]. The surface charge of liposomes has been reported to affect the liposome uptake in *in vitro* cell cultures. Generally, positively-charged liposomes are taken up by APCs to a much higher degree than negatively-charged or neutral liposomes [20,21,22]. Thus, positively-charged dioleolyldimethylammonium propanediol:dioleolyl phosphatidylcholine (DODAP:DOPC)-liposomes showed the highest fraction of uptake in J774 macrophages, as compared to negatively-charged dioleolyl phosphatidylserine (DOPS):DOPC-liposomes and neutral DOPC-liposomes [21]. Furthermore, *in vitro*-generated human dendritic cells (DCs) incubated with positively-charged dimyristoyl trimethylammonium-propane:dimyristoyl phosphatidylcholine (DMTAP:DMPC):Chol-liposomes (zeta-potential (zp) = +44.2 mV) took up the liposomes to a much higher degree than when they were incubated with negatively-charged dimyristoyl phosphatidylglycerol (DMPG):DMPC:Chol-liposomes (zp = −54.2 mV) and dimyristoyl phosphatidylserine (DMPS):DMPC:Chol-liposomes (zp = −50.0 mV) [22].

The surface charge has also been shown to have a pronounced effect on the antibody response. The significantly higher cellular uptake of positively-charged, non-sized SA:EPC:Chol-liposomes (zp = +8.85 mV) as compared to neutral, non-sized EPC:Chol-liposomes (zp = −5.04 mV) or negatively-charged, non-sized dimyristoyl phosphatidic acid (DMPA):EPC:Chol-liposomes (zp = −18.6 mV) in murine peritoneal-derived macrophages correlated with a much higher induction of antibodies following s.c. immunization with OVA [20]. In a model using an avirulent Semliki Forest virus strain adjuvanted with liposomes administered intraperitoneally, only the positively-charged, non-sized octadecylamine (ODA):DPPC:Chol-liposomes and negatively-charged, non-sized PA:DPPC:Chol-liposomes were able to induce a significant antibody response, whereas no effect was observed with the neutral, non-sized DPPC:Chol-liposomes [30]. The surface charge of the liposomes also influences their interactions with endogenous tissue components, such as proteins, enzymes and cells, eventually affecting the retention at the SOI, their draining kinetics and localization in the draining organs [31,32,33]. The positively-charged CAF01 (zp = +50 mV) was compared to neutral distearoyl phosphatidylcholine (DSPC):TDB-liposomes (zp = −8 mV) following i.m. immunization. The neutral liposomes drained from the SOI significantly faster than CAF01, despite the larger particle size of 1620 nm, as compared to CAF01 (475 nm) [29]. Only CAF01 was capable of inducing a cell-mediated immune (CMI) response as measured by the expression of IFN-γ and IL-17 in activated T cells, which could be because of the retention of CAF01 at the SOI, but also the ability of DDA to induce a pro-inflammatory environment at the SOI with influx of innate immune cells and APCs [29,34]. Even though cationic liposomes are generally more immunogenic than anionic and neutral liposomes, as shown above, Badiee *et al.* found that neutral, 1.1 µm-sized DPPC:Chol-liposomes were superior in reducing the parasitic burden in a Leishmania challenge model, followed by positively-charged, 1.46 µm-sized DDA:DPPC:Chol-liposomes and negatively-charged, 1.23 µm-sized DCP:DPPC:Chol-liposomes [35].

### 2.3. Effect of Surface Modification

Designing stealth liposomes via, e.g., PEGylation is an alternative strategy for modifying the surface properties of liposomes, which can affect the biodistribution pattern of administered vaccine particles. Hydrophilic polyethylene glycol (PEG) moieties are usually applied for surface modification, either by covalent attachment to anchor lipids in the membranes or via different linkers that are specifically cleaved by changes in pH, enzymatic stimuli or reducing agents, removing the PEG moieties from the liposome surface [36]. In one study, increasing the PEG density from 0 to 7 mol% on positively-charged DOTAP:DPPC:Chol-liposomes decreased the *in vitro* uptake by human pancreatic carcinoma cells [37]. This was not observed with positively-charged dioleyldimethylammonium chloride (DODAC):DOPE-liposomes in another study; however, the ability of the liposomes to transfect cells with plasmid DNA (pDNA) was inversely correlated with the PEG chain length (MW = 220, 1450 and 3400) at 5 mol% [38]. I.m. administration of PEGylated CAF01 (10 and 25 mol% DSPE-PEG_2000_) resulted in an increased drainage from the SOI as compared to non-PEGylated CAF01 [39,40]. The IFN-γ-driven T-cell response was significantly higher following immunization with non-PEGylated CAF01 compared to PEGylated CAF01, whereas the IL-5 response was highest with the PEGylated CAF01 [39,40]. Thus, PEGylation of liposomes can influence the type of the induced immune response.

### 2.4. Effect of Lipid Bilayer Fluidity

The lipid bilayer of the liposomes can be in different physical phases depending on the specific lipid composition and temperature. Lipids with a main phase transition temperature (T_m_) below 37 °C will be in a liquid-crystalline state (fluid-disordered phase) in the body, while they will be in a gel state (solid-ordered phase) if the T_m_ is above 37 °C.

The physical state of the bilayer (gel state or liquid-crystalline state) might affect endocytosis, intracellular trafficking and processing of the vaccine components, which in turn may influence the immune responses. *In vitro* studies comparing fluid-disordered phase dioleoyl phosphatidylglycerol (DOPG):DOPC:DOPE:Chol-liposomes with solid-ordered phase distearoyl phosphoethanolamine:distearoyl phosphatidylglycerol (DSPE:DSPG):DSPC:Chol-liposomes showed that the fluid-disordered phase liposomes delivered the antigen to the major histocompatibility complex (MHC)-I processing compartments to a higher degree than the solid-ordered liposomes [41]. In *in vivo* studies, the solid-ordered phase (CAF01) liposomes was compared to the unsaturated analog DODAC combined with TDB, resulting in fluid-disordered liposomes at body temperature. CAF01 formed a depot at the SOI, whereas the DODAC:TDB-liposomes quickly escaped the SOI and were detected in significantly higher amounts in the dLN shortly after immunization. The biodistribution of the attached antigen did not differ depending on the adjuvant; however, only CAF01 was capable of inducing a T-cell response [42].

The immunopotentiating effect of liposomes with different T_m_ was evaluated in hamsters, where DSPC (T_m_ 54 °C), DPPC (T_m_ 41 °C) and DMPC (T_m_ 23 °C), all prepared as liposomes with Chol, were compared [23]. No differences in the antigen loading efficiency or humoral responses were observed, but the DSPC:Chol-liposomes were superior in inducing CMI responses, which was attributed to increased adjuvant stability *in vivo* [23]. A similar trend on the CMI responses was observed in a study comparing DSPC, DPPC and EPC (T_m_ < 0 °C) combined with Chol. The DSPC- and DPPC-based liposomes caused the largest reduction in the infection burden in a Leishmania parasite challenge, while the EPC-based liposomes were not different from the unadjuvanted antigen [24]. Thus, the fluidity of the liposome bilayer affects the biodistribution of the adjuvant and the CMI responses that are induced upon vaccination.

## 3. Pathogen-Derived Immunostimulators Are Ligands for Pattern-Recognition Receptors

**Figure 1:**
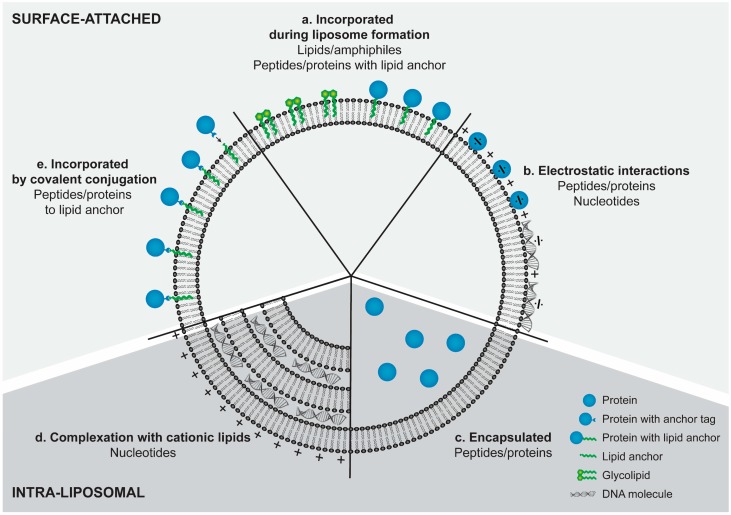
Different strategies can be employed for incorporating antigens and immunostimulators into liposomes depending on the type and purpose of the molecules in question. The formulation and structure of the liposomes allows the incorporation of different molecules by different strategies into the same liposomes and the precise tailoring of the adjuvant towards a certain target. (**a**) Hydrophobic molecules and lipids can be incorporated into the lipid bilayer by addition to the dissolved lipids prior to dry film formation. (**b**) Peptides/proteins and nucleotides can be electrostatically adsorbed to oppositely-charged lipids on the surface of liposomes. (**c**) Peptides and proteins can be encapsulated into the aqueous interior of the liposomes, e.g., by the dehydration-rehydration method. (**d**) Nucleotides can be complexed with cationic lipids being embedded between multiple lipid bilayers. (**e**) Post-liposome manufacture attachment of peptides and proteins can be achieved by covalent conjugation to functionalized lipid anchors.

The antigens in subunit vaccines are predominantly based on peptides and proteins, while the immunostimulators may be derived from all classes of molecules; DNA, RNA, lipids, sugars, small molecules, peptides and proteins. These molecules are vastly different and therefore different strategies for association with the liposomes are required. Due to the versatility of liposomes, it is possible to incorporate all types of immunostimulators into the same liposome dispersion (Figure 1).

**Figure 2:**
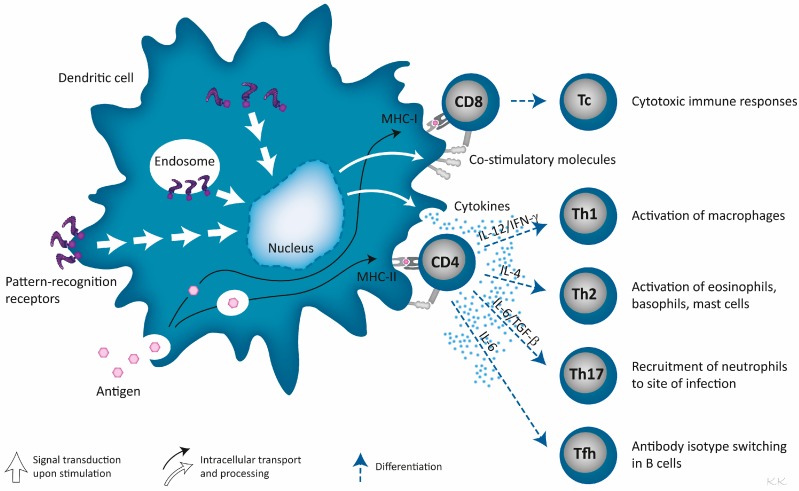
Major immunological responses derived by the activation of DCs through PRRs, situated on the cell surface, in the cytosol or in the endosomes. The antigen is phagocytosed, processed and presented on MHC-I via cross-presentation or on MHC-II to antigen-specific CD8^+^ and CD4^+^ T cells, respectively. Activation of the DC upregulates co-stimulatory molecules and secretion of cytokines that provide activation signals to the antigen-specific T cells. Activation of CD8^+^ T cells may require additional IL-2 from CD4^+^ T cells (not shown) to become cytotoxic T cells. Activated CD4^+^ T cells differentiate to distinct effector populations depending on the received cytokine signals; Th1 requires secretion of IL-12/IFN-γ; Th2 requires IL-4; Th17 requires IL6/TGF-β; and follicular T-helper cells (Tfh) requires IL-6.

Immunostimulators are ligands of PRRs, such as the Toll-like receptors (TLRs), NOD-like receptors (NLRs), RIG-I-like receptors (RLRs) and C-type lectin receptors (CLRs). These receptors are expressed on the surface, in the endosomes or in the cytosol of APCs, where each type of receptor is capable of recognizing characteristic bacteria- and virus-derived molecular structures, the so-called pathogen-associated molecular patterns (PAMPs) [43,44,45,46]. Activation of PRRs, by their respective ligands, generally sets off pro-inflammatory responses and/or type I IFNs. However, differences in the signaling pathways induced by the different receptors can skew the immune response towards different outcomes, *i.e.*, towards Th1 or Th2 responses [46,47] (Figure 2).

Thus, the choice and combination of immunostimulators play an important role in controlling the type of immune response induced following vaccination though other factors, such as the administration route and the physicochemical properties of the liposomes, which also have an impact on the resulting immune response. The skewing of the immune responses might reflect the origin of the respective ligands, *i.e.*, PRRs recognizing ligands of bacterial origin often induce a Th1 response, which is suitable for fighting certain bacterial infections [48]. Similarly, double-stranded (ds)RNA derived from virus induces cytotoxic T-lymphocyte (CTL) immune responses, capable of combatting a virus infection [49].

The different receptors recognizing PAMPs are located in different parts of the cells; surface associated (e.g., TLR1, -2, -4, -5, -6, CLRs), in the endosomes (e.g., TLR3, -7, -8, -9) or in the cytoplasm (e.g., NLRs, RLRs) [43,45,46]. The cellular compartmentalization reflects the localization of the ligands; the ligands of the surface-associated PRRs are expressed on the surface of the pathogen, while the ligands of the endosomal TLRs are, e.g., nucleotides [46,50].

## 4. Incorporation Strategies for Amphiphilic Lipids and Hydrophobic Compounds

Several immunostimulating molecules are lipids or hydrophobic molecules and, thus, suitable for direct incorporation into the liposomal bilayer. Typically, the lipids used in the preparation of liposomes are dissolved in an organic solvent, and a dry lipid film is then formed upon evaporation of the solvent according to a method originally devised by Bangham *et al.* [51]. Hydrophobic immunostimulators can be added to the organic phase and incorporated into the lipid bilayer via the dry lipid film method (Figure 1a).

Lipid-based immunostimulators that are incorporated into the liposomal bilayer by addition to the dissolved lipids include molecules derived from the cell walls of bacteria. One such immunostimulator is the TLR4 agonist monophosphoryl lipid-A (MPL), which is a derivative of lipopolysaccharide (LPS). The presence of MPL induced higher antibody and CMI responses following immunization in mice as compared to liposomes without MPL [52,53]. LPS and MPL are pyrogenic when administered freely, but incorporation of LPS and MPL into liposomal dispersions reduces these side effects [54]. The synthetic analogs of lipids derived from the cell wall of *M. bovis* BCG, TDB and monomycoloyl glycerol (MMG) have been extensively studied as immunostimulators in vaccine adjuvants [55,56,57,58,59]. The immunogenic effects of CAF01 and CAF04 (DDA:MMG-liposomes) are similar, inducing antibody and strong Th1 and Th17 CMI responses in mice, which are not achieved with neat DDA liposomes [55,56,58,59,60]. CAF01 has been shown to elicit similar immune responses when administered with different antigens (the model antigen OVA, the chlamydia vaccine candidate MOMP, the TB vaccine candidate Ag85b-ESAT-6, the malaria vaccine candidate MSP1-19 and trivalent influenza vaccine [TIV]) [56,60]. Beside the role as immunostimulators, TDB and MMG act as colloidal stabilizers for the liposomes, as neat DDA liposomes are not stable in formulation [55,59].

Covalent attachment of hydrophilic compounds to a lipid anchor enables the incorporation of these compounds into the lipid bilayer during the formulation of the liposomes (Figure 1a). Mannose receptors, which are CLRs, are present on DCs and facilitate endocytosis upon activation resulting in improved DC activation [61,62]. Tri- and di-mannose-conjugated lipids can be incorporated directly into the liposome bilayer during lipid film formation, thus enabling activation of the mannose receptor in the immediate vicinity of the liposomes [61,63]. The uptake of mannosylated liposomes was enhanced *in vitro* [61], while earlier, systemic IgG antibodies were induced in a mouse model [63] as compared to the corresponding formulations without mannose lipid.

Lipid anchoring of the antigen is a strategy in which pre-conjugated antigen-lipid complexes are included into the lipid solution prior to dry film formation [64,65,66] (Figure 1a). In one study, tumor-specific E7 peptide-antigen was attached to cationic liposomes by a lipid anchor, and the anti-tumor effect was compared to encapsulation of the non-lipidated E7 (prepared by rehydration of the lipid film with aqueous buffer containing the antigen). The lipid-anchored peptide elicited a greater anti-tumor effect and improved CD8^+^ T-cell responses than the non-lipidated, encapsulated peptide [65]. In one study, the vaccine candidate, peptide antigen, MUC1, induced antibody responses only when attached to the liposomes with a lipid anchor, which were absent when the antigen was encapsulated in the aqueous interior of the liposomes. Both formulations induced similar levels of a Th1-directed CMI response [66]. Induction of antibody responses requires interaction between soluble or APC surface-attached antigen and receptors on the surface of B-cells, as well as help by CD4^+^ T cells, events which occur in the lymph nodes and the spleen [67,68]. However, in another study, comparing surface-attached antigen and entrapped antigen, no difference in the antibody responses was reported [69].

High incorporation efficiencies can be achieved for several different lipophilic compounds when they are incorporated into the liposomal membranes by addition to the dissolved lipids. The incorporation ratios of retinoic acid and lipoprotein in liquid-crystalline state liposomes were 98% to 100% and 75% to 80%, respectively [70]. Similar loading efficiencies of 79% to 81% were achieved when incorporating the synthetic TLR4 agonist glucopyranosyl lipid adjuvant (GLA) into liquid-crystalline state liposomes [71]. A loading efficiency of 90% to 95% was achieved with a lipopeptide antigen, which far exceeded the loading efficiency of ~25% achieved when incorporating the soluble form of the antigen into the aqueous interior. The lipid was attached to the peptide via an amino acid spacer to avoid interference with the epitope [65]. Lipidation of peptides or protein can affect the presentation and processing of the antigen to APCs, and the method for lipidation should be considered carefully. Thus, compared to the incorporation by the rehydration method or electrostatic association, incorporation of lipophilic compounds into the lipid membrane generally results in high encapsulation efficiencies, as the molecules become an integral part of the liposomal membrane.

## 5. Incorporation Strategies for Nucleic Acids

### 5.1. RNA/DNA-Based Immunostimulators

Several immunostimulators are nucleic acids, e.g., dsRNA and DNA with unmethylated CpG motifs. dsRNAs are virus-specific agonists of TLR3, which is highly expressed in cross-presenting DCs and induces a CTL immune response when activated [49,72]. The most commonly studied TLR3 ligand is polyinosinic:polycytidylic acid (poly(I:C)), a synthetic analogue of dsRNA. The implications and considerations of the formulation of poly(I:C)-containing adjuvants were reviewed in detail by Hafner *et al.* [73]. DNA of bacterial origin contain CpG motifs, which are recognized by TLR9. A great variety of cells of the immune system, including B cells, NK cells, DCs and macrophages, express TLR9 and are activated upon stimulation with CpGs [74]. Therefore, CpG incorporated into a subunit vaccine acts as an immunostimulator and enhances and directs the type of immune response. The resulting immune response varies depending on the specific type of CpG motif applied and depending on the target cells [75]. When using CpG as vaccine adjuvants, synthetic CpG oligodeoxynucleotides (CpG-ODNs) are the preferred choice [74].

Since nucleic acids are hydrophilic, they are incorporated into the liposomes during or after the rehydration of the dry lipid film. One method for complexation of nucleic acids with cationic lipids is the dehydration-rehydration method, where the nucleic acids and liposomes are mixed, freeze-dried and subsequently rehydrated in a controlled manner [40,76,77,78,79]. Another method is the double-emulsion method in which an initial water-in-oil emulsion is formed with the nucleic acids in the water phase and the lipids in the organic phase, followed by the addition of a second aqueous phase forming a water-in-oil-in-water emulsion. Finally, the organic phase is evaporated resulting in encapsulation of the nucleic acids inside the multilamellar liposomes [80] (Figure 1d). The use of positively-charged lipids is a preferred method to increase the incorporation efficiencies of the nucleic acids into liposomes [81]. Incorporation of CpG-ODN into liposomes via the dehydration-rehydration method showed that the encapsulation efficiency was highest in cationic and cationic-stealth liposomes (60% to 90% of CpG-ODN encapsulated), as compared to neutral, anionic and stealth liposomes (33% to 54% of CpG-ODN encapsulated) [76], while a loading efficiency of 99% was achieved by the rehydration method with CpG and cationic liposomes [82].

The nucleic acids can also be attached to the surface of preformed liposomes via electrostatic interactions with positively-charged lipids in the liposomal bilayer [81,83,84,85,86,87] (Figure 1b). Both the solid-ordered CAF01 and CAF04 and the liquid-crystalline, cationic DOTAP:DOPC-liposomes loaded with poly(I:C) via electrostatic surface attachment induce strong CD8^+^ T-cell responses against several different antigens (OVA, E7, the cancer antigen TRP-2, the TB vaccine antigens TB10.3-P1 and H56 and the HIV vaccine antigen Gag p24) in mice [83,84,85,87]. Poly(I:C) adsorbed to zwitterionic liposomes caused a significant influx of neutrophils and monocytes in the lymphatic vessels following s.c. immunization of sheep as compared to administration of the same vaccine without poly(I:C) [88]. Stepwise addition of poly(I:C) can be required when adsorbing it to the surface of cationic liposomes to avoid aggregation of the liposome particles [83,85,87]. CpG adsorbed to the surface of cationic, liquid-crystalline liposomes resulted in increased CD4^+^ and CD8^+^ T-cell responses and increased survival in a mouse cancer-challenge model, as compared to liposomes without CpG [86], while IFN-γ-producing CD4^+^ and CD8^+^ T cells were induced by CpG-conjugated OVA incorporated into the liposomes via the dehydration-rehydration method [78]. TLR3 and TLR9 are both located in the endosomes, and therefore, the induced immune responses do not depend on whether the immunostimulators are attached to the surface or in the interior of the liposomes.

Administration of unadjuvanted poly(I:C) causes strong inflammatory immune responses with increasing production of IL-6 shortly after the immunization, which is greatly reduced with adsorption of poly(I:C) to liposomes [80]. The use of liposomes as a delivery system thus serves to ensure co-delivery of immunostimulators and antigen, while at the same time minimizing the detrimental effects of poly(I:C).

TLR7 recognizes single-stranded RNA, but the most used agonists are the synthetic imidazoquinoline derivatives, imiquimod and resiquimod [89]. Imiquimod is a poorly soluble compound, but its solubility is increased at low pH; therefore, incorporation was achieved by using the rehydration method using an acidic lactic acid buffer followed by sonication and extrusion. Different encapsulation efficiencies were observed with liposomes of various surface charges [71]. Cationic liposomes had the lowest encapsulation efficiency (0%), whereas neutral and anionic liposomes had slightly higher encapsulation efficiencies (6% to 7%) [71]. Incorporation of imiquimod into cationic liposomes by addition to the organic phase with dissolved lipids prior to dry lipid film formation resulted in an incorporation efficiency of 100% [82]. Thus, the incorporation strategy dramatically influences the loading efficiency of imiquimod in liposomes. By exploiting the improved solubility of the compound in an organic solvent, the encapsulation efficiency could be greatly enhanced.

### 5.2. DNA–Antigen Vectors

pDNA vaccines are explored as an alternative to the peptide or protein antigen-based vaccines. In this vaccination strategy, pDNA encoding for one or more pathogen-specific antigens is delivered to target cells, and the proteins are then expressed. Thus, the antigen is produced directly in the cell, and the epitopes are then presented on MHC-I or the whole protein antigen is secreted from the cell [90].

The preferred formulation method is encapsulation of the pDNA into cationic liposomes [28,91,92,93], with the purpose of protecting the pDNA from degradation by deoxyribonucleases in the interstitial fluids [93] and improving the transfection ratio [92]. The pDNA entrapment efficiency thus increased significantly when the liposomes contained cationic lipids, enabling pDNA:lipid-complexation [79] (Figure 1d). Gel state and liquid-crystalline state liposomes showed no difference in pDNA entrapment efficiencies, but an enhanced transfection efficiency was observed with liquid-crystalline liposomes. This effect was attributed to enhanced interaction of the liposomes with the endosomal bilayer, eventually resulting in endosomal escape [92]. An often-used method for pDNA encapsulation with lipid complexation is the above-mentioned dehydration-rehydration procedure [28,92,93]. Microfluidization is a relatively novel manufacturing technology, which has also been used for the complexation of pDNA with cationic, liquid-crystalline liposomes [94]. With this method, pDNA solubilized in aqueous buffer is led in between two perpendicular streams of lipids dispersed in an organic solvent, and the complex is formed by dilution of the buffer into the organic solvent [16,94].

## 6. Incorporation Strategies for Peptides and Proteins

The antigen in a subunit vaccine is often a protein or peptide derived from the pathogen of interest. Co-delivery of antigen and adjuvant is usually necessary for inducing a sufficient immune response [9]. Therefore, several different methods have been developed to incorporate antigen(s) into the liposome-based adjuvants. Different strategies can be exploited for incorporation of antigen(s) into a liposomal adjuvant: (i) covalent conjugation via a lipid anchor; (ii) non-covalent attachment; (iii) encapsulation in the aqueous interior; (iv) electrostatic complexation with oppositely-charged lipids; and (v) adsorption to the liposome surface [95].

Antigens were encapsulated by using the dehydration-rehydration method with high encapsulation efficiencies of 80% to 90% [77]. Similarly, encapsulation efficiencies of >90% were achieved for IL-2 in DMPC:DMPG-liposomes via the dehydration-rehydration method. However, the authors speculated that the high encapsulation efficiencies were achieved because the IL-2 was partially bound by hydrophobic and electrostatic interactions [96]. Thus, reported high encapsulation efficiencies may also cover other modes of interactions. A lower encapsulation efficiency of 25% was reported following encapsulation of the E7 peptide antigen to DOTAP-based liposomes [65] (Figure 1c). Electrostatic adsorption of the antigen to the liposome adjuvant is a strategy that enhances co-delivery of the adjuvant and the antigen to the APCs (Figure 1b). The degree of antigen adsorption to the liposomes depends on the attractive electrostatic forces between the components. Thus, more protein antigens with isoelectric points (pIs) below 7.4 adsorb onto cationic liposomes at pH 7.4, as compared to proteins with a pI above 7.4 or with neutral liposomes [97,98]. The addition of proteins with pIs below 7.4 to positively-charged liposomes induced the aggregation of the liposomes. However, at high protein concentrations (far exceeding vaccine dose concentration), the liposomes are stabilized by the formation of a protein corona on the surface [97]. In one study, the degree of antigen adsorption is a contributing factor to the efficacy of subunit vaccines. Only cationic liposomes with antigen adsorbed to the surface induced a CMI response, possibly due to increased retention of the antigen at the SOI increasing the exposure to APCs, as compared to neutral liposomes with no antigen adsorption [29].

Antigens can also be covalently attached to preformed liposomes. This strategy requires the incorporation of a functionalized lipid anchor into the liposome membrane, followed by attachment of a functionalized protein or peptide via an activation step ensuring conjugation, e.g., by a maleamide-thiol reaction or a NTA_3_-DTDA-His reaction [69,99,100,101] (Figure 1e). Covalent conjugation of the antigen to neutral liposomes resulted in increased antibody responses as compared to the unconjugated antigen mixed with the liposomes [99], while covalent conjugation resulted in significantly higher CMI responses, but no differences in the antibody response, as compared to encapsulated antigen [69]. Furthermore, TLR5 peptide ligands covalently conjugated to NTA_3_-DTDA in stealth liposomes showed enhanced TLR5-mediated cellular uptake *in vitro* [101].

Post-manufacture surface attachment of proteins and peptides, whether by electrostatic interaction or covalent binding, requires the proteins or peptides in question to be relatively water soluble. However, it may be required to incorporate hydrophobic and poorly water soluble proteins and peptides. The highest encapsulation efficiencies were achieved with the urea method (40% encapsulated), in which the dried lipid film was rehydrated in a urea and peptide-containing buffer, followed by sonication and extrusion. Lower encapsulation efficiencies were achieved with the ethanol destabilization method, where the dried lipid film was rehydrated in an ethanol:water buffer with the addition of peptide dissolved in dimethyl sulfoxide (DMSO) or by adding the peptide dissolved in DMSO to the lipids dissolved in an organic solvent (30% and 25% encapsulated, respectively) [86].

By using covalent, post-manufacture attachment of proteins and peptides, more possibilities are available when designing the liposomes and in the choice of peptide or protein. When using electrostatic attachment, opposite electric charges are required, which might not be optimal in the desired formulation.

The loading strategy chosen for a given antigen, or protein- or peptide-based immunostimulator depends on the function of the protein or peptide and the physicochemical properties of the liposomes, proteins and peptides. Since these parameters affect the need for, as well as the possibilities of, e.g., surface attachment, the loading strategy may have a significant impact on the quality of the formulation and the obtained immune response.

## 7. Clinical Experience with Liposome-Based Adjuvants

Intensive development efforts have resulted in a number of different liposome-based adjuvant candidates as part of marketed or clinically-tested vaccines against several different pathogens during the past 20 years (Table 1). The clinically-tested liposome-adjuvanted vaccine formulations were generally well tolerated by the study participants. Prophylactic vaccines against several different pathogens have been tested, and representative examples of these are presented below. In these studies, the liposome-adjuvanted vaccine formulations may serve to induce an immune response against a pathogen-specific antigen or to improve the quality of the immune response already achieved using a marketed vaccine.

Commercial influenza split vaccines were adjuvanted with DMPG:DMPC–liposomes loaded with IL-2 (this combined vaccine was named INFLUSOME-VAC) and compared to the unadjuvanted split vaccine in young, healthy adults and elderly, nursing-home residents. In both age groups, vaccination with INFLUSOME-VAC resulted in increased hemagglutinin (HA) titers as compared to the unadjuvanted vaccine [106,107]. In contrast, no difference in the serologic responses were observed in a trial in seropositive elderly, after vaccination with H1N1 split virus adjuvanted with DMPC:Chol-liposomes as compared to the unadjuvanted vaccine [102]. DPMC:DMPG:Chol:MPL-liposomes were safe in healthy adults with induction of bactericidal antibodies in an *N. meningitides* vaccine trial. However, the vaccine strategy was discontinued with no reason provided [112]. In a phase I trial (NCT00922363), a subunit vaccine against *M. tuberculosis* consisting of the H1 antigen adjuvanted with CAF01 was found to be safe and induced long-lasting T-cell responses, though no antibody responses were observed in healthy adults [116]. The liposomal adjuvant system AS01, containing MPL and QS21, an NLR inflammasome-inducing, saponin derivative [117], has been evaluated with the malaria antigen RTS,S (NCT00307021, NCT00075049, NCT00197054) and the *M. tuberculosis* antigen M72 (NCT00621322) [108,109,110,115]. In the malaria vaccine studies, the safety of RTS,S adjuvanted with AS01 was compared to the antigen adjuvanted with the emulsion-based adjuvant AS02, which also contains MPL and QS21. In children and healthy adults, both adjuvants were safe, but AS01 was found to induce slightly higher antibody titers [108,109,110]. In a study with the M72 antigen in healthy, purified protein derivative (PPD)-positive (BCG vaccinated) adults, both AS01 and AS02 induced CMI and humoral responses. However, AS01 was recommended for further studies [115]. The effect of DPPC:DCP:Chol-liposomes upon intranasal immunization for the induction of a mucosal IgA antibody response was tested in a study against *S. mutans*. Significantly higher nasal IgA responses were observed, though no difference was observed in the serum and saliva responses [104]. Epaxal, a marketed hepatitis A vaccine, consists of antigens surface-attached onto phosphatidylcholine:phosphatidylethanolamine-fatty acid (PC:PE)-liposomes containing influenza HA and neuraminidase (NA) (also known as virosomes). The vaccine was administered twice at 12 months intervals and 100% of the participants seroconverted following the second immunization (*n* = 117) [103].

Liposomes have also been evaluated as adjuvants in therapeutic vaccine settings. In one trial, mite allergic asthma patients were vaccinated with extract from dust mite bodies adjuvanted with DPPC:Chol:tocopheryl acid succinate liposomes. Subjects immunized with both extract and liposomes were better protected than the control group immunized with only liposomes, in terms of reduced reaction to allergen challenge [105]. In a study with breast cancer patients, a recombinant dHER2 protein was incorporated into the liposomal adjuvant AS15 containing MPL, QS21 and CpG. The vaccine was generally well tolerated, but the number of participants (*n* = 12) was too small for statistical conclusions on the effect [113]. The vaccine Tecemotide, L-BLP25, was tested in a phase III study of stage III non-small cell lung cancer (NCT01015443). L-BLP25 consists of a lipopeptide antigen, BLP25, incorporated into DMPG:DPPC:Chol:MPL-liposomes administered by s.c. immunizations [111]. However, the study was terminated due to no effect on the primary endpoint, overall survival or the secondary endpoints (progression-free survival, time to progression and time to treatment failure) in a phase II study [118]. CAF01 was also tested with a cocktail of peptides in a study against HIV (NCT01141205). Whereas the vaccine was well-tolerated and 6/14 participants presented new responses to T-cell epitopes in the vaccine, no change in viral load was detected [114].

The referred clinical trials address liposome dispersions of highly different composition illustrating the versatile potential of liposomes as an adjuvant. However, as can be seen, the majority of the dispersions are phospholipid-based liposomes with MPL as the immunostimulator. This may be due to the strict control of substances that are approved for human use, implying that compounds that have been approved for human use once might be preferred for further development and clinical trials.

## 8. Future Perspectives: Challenges in the Further Development of Liposome-Based Adjuvants

In the present review, we have illustrated the versatility of liposomes in terms of incorporation of antigens and immunostimulators. This allows the design of liposomal adjuvants optimally tailored for the target, be it prophylactic or therapeutic vaccines against pathogens, cancer or allergens.

In the vaccine setting, the challenge is to deliver the antigen(s) and the immunostimulator(s) to the correct cells of the immune system. Depending on the desired immune response of the vaccine, be it a humoral or CMI response, or even a regulatory T-cell response, different types of cells must be targeted and activated. An example are TLRs, which are differentially expressed by subsets of APCs, thus shaping the capability of the APCs to induce specific immune responses in response to stimulation [44,45]. Furthermore, the stability of liposome-adjuvanted vaccines should be addressed early in the development of the vaccine. Since the vaccines are likely to contain components from different molecular classes, e.g., the chemical, the colloidal and the physical stability of the different components should be considered.

The intricacies of the immune system are still being explored, and new subsets of DCs, T cells and other cells of the immune system are continuously being discovered and described. New opportunities in the development of subunit vaccines can be explored as novel target-cell populations are being discovered. The many different target cells can require widely different modes of activation in terms of immunostimulators and presentation of the different components of the vaccine. The challenge will be to develop, in a systematic way, robust subunit vaccine formulations with adjuvant systems that contain all of the required immunostimulators presented correctly to the target cells. The quality-by-design approach using the rational design of both antigen and adjuvant, while implementing risk assessment and design of experiments, is in this context a useful tool for optimizing such complex formulation and manufacturing processes [3,119]. Liposomes are good candidates for developing such multifunctional adjuvants using these principles, as they can be tailored by the choice of lipid components and by the preparation method, while several different types of immunostimulators can be incorporated into the same adjuvant system.

## Figures and Tables

**Table 1:** Representative clinical trials using liposome-based adjuvants in vaccines. Representative examples of clinical trials evaluating liposome-based adjuvants in vaccines in the past 20 years. The targets for the liposome-based adjuvant vaccines are both prophylactic and therapeutic, covering a range of pathogens and diseases. Epaxal has been approved for human use. NCT: National Clinical Trial number.

Year Published	Target	Lipids in Liposomes	Immunomodulators/Potentiators	Antigen	Prophylactic/Therapeutic	Phase	NCT	Refs.
1995	Influenza	DMPC:Chol	-	H1N1 Split virus	P	I	-	[102]
1997	Hepatitis A	Phospholipids	HA, NA	Inactivated hepatitis A virus particles	P	I	-	[103]
1999	Streptococcus mutans	DPPC:DCP: Chol	-	C-GTF	P	I	-	[104]
2002	Mite allergy: *D. pteronyssinus*	DPPC:Chol	Tocopheryl acid succinate (vitamin E)	Mite body extract	T	I	-	[105]
2003	Influenza	DMPC:DMPG	IL-2	H1N1 Split virus	P	I/II	-	[106,107]
2009	Malaria	-	MPL, QS21 (AS01)	RTS,S	P	I/II	NCT00307021, NCT00075049, NCT00197054	[108,109,110]
2011	Lung cancer	DMPG:DPPC:Chol	MPL	BLP25	T	III	NCT01015443	[111]
2012	Neisseria meningitidis	DMPG:DMPC:Chol	MPL	Outer membrane proteins and deacetylated lipooligosaccharide	P	I	-	[112]
2012	Breast cancer	-	MPL, QS21, CpG (AS15)	dHER2 protein	T	I	-	[113]
2013	HIV	DDA	TDB (CAF01)	Cocktail of peptides	T	I	NCT01141205	[114]
2013	Mycobacterium tuberculosis	-	MPL, QS21 (AS01)	M72	P	I	NCT00621322	[115]
2014	Mycobacterium tuberculosis	DDA	TDB (CAF01)	H1 protein	P	I	NCT00922363	[116]

## Abbreviations

The following abbreviations are used in this manuscript:

APC: Antigen-presenting cell
Chol: Cholesterol
CLR: C-type lectin receptor
CMI: Cell-mediated immunity
CpG-ODN: CpG oligodeoxynucleotide
CTL: Cytotoxic T-lymphocyte
DC: Dendritic cell
DCP: Dicetyl phosphate
DDA: Dimethyldioctadecylammonium bromide
DMPA: Dimyristoyl phosphatidic acid
DMPC: Dimyristoyl phosphatidylcholine
DMPG: Dimyristoyl phosphatidylglycerol
DMPS: Dimyristoyl phosphatidylserine
DMSO: Dimethyl sulfoxide
DMTAP: Dimyristoyl trimethylammonium-propane
DODAC: Dioleyldimethylammonium chloride
DODAP: Dioleoyl dimethylammonium propanediol
DOPC: Dioleolyl phosphatidylcholine
DOPE: Dioleoyl phosphoethanolamine
DOPS: Dioleolyl phosphatidylserine
DOTAP: Dioleoyl trimethylammonium-propane
DPPC: Dipalmitoyl phosphatidylcholine
DSPC: Distearoyl phosphatidylcholine
DSPE: Distearoyl phosphoethanolamine
DSPG: Distearoyl phosphatidylglycerol
dsRNA: Double-stranded RNA
DT: Diphtheria toxoid
EPC: Egg-phosphatidylcholine
EPG: Egg-phosphatidylglycerol
GLA: Glucopyranosyl lipid adjuvant
HA: Hemagglutinin
IFN: Interferon
i.m.: Intramuscular
LPS: Lipopolysaccharide
MHC: Major histocompatibility complex
MMG: Monomycoloyl glycerol
MPL: Monophosphoryl lipid-A
NA: Neuraminidase
NLR: NOD-like receptor
OVA: Chicken egg ovalbumin
PA: Phosphatidic acid
PAMP: Pathogen-associated molecular pattern
PC: Phosphatidylcholine
PDI: Polydispersity index
pDNA: Plasmid DNA
PE: Phosphatidylethanolamine
PEG: Polyethylene glycol
pI: Isoelectric point
Poly(I:C): Polyinosinic:polycytidylic acid
PPD: Purified protein derivative
PRR: Pattern recognition receptor
RLR: RIG-I-like receptor
SA: Stearylamine
s.c.: Subcutaneous
SOI: Site of injection
TDB: Trehalose-6,6´-dibehenate
Th: T-helper cell
Tfh: Follicular T-helper cell
TLR: Toll-like receptor
T_m_: Main phase transition temperature
Zp: Zeta-potential
